# Supplementary material for: Nutrient limitation determines the fitness of cheaters in bacterial siderophore cooperation
Source: Nat Commun. 2017 Aug 10;8:230. doi: 10.1038/s41467-017-00222-2 (PMC5550491; doi:10.1038/s41467-017-00222-2)
Supplement: Supplementary file 1 — Supplementary Information [file 41467_2017_222_MOESM1_ESM.pdf]

File name: Supplementary Information
Description: Supplementary figures, supplementary tables, supplementary note, supplementary methods and supplementary references.

File name: Peer review file
Description:

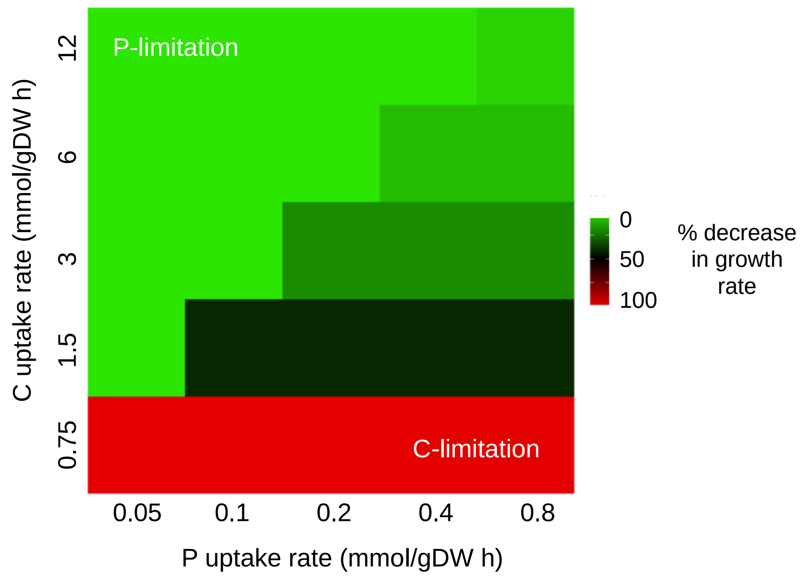

Supplementary Fig. 1. Relative C and P availabilities determine the impact of PVD secretion on cellular biomass production. The heat map shows the secretion-dependent percent-decrease in growth rate as a function of C and P-uptake rates. For each nutrient combination, growth rates were compared in the presence and in the absence of secretion. The PVD secretion rate was set to a constant value (0.050 mmol (g DW h)$^{-1}$, equal to 0.068 g (g DW h)$^{-1}$), such that it reduces the growth rate by almost 100% at the lowest nutrient uptake rates. Relatively low C and high P uptake rates yield C-limiting conditions, whereas relatively low P and high C uptake rates yield P-limiting conditions.

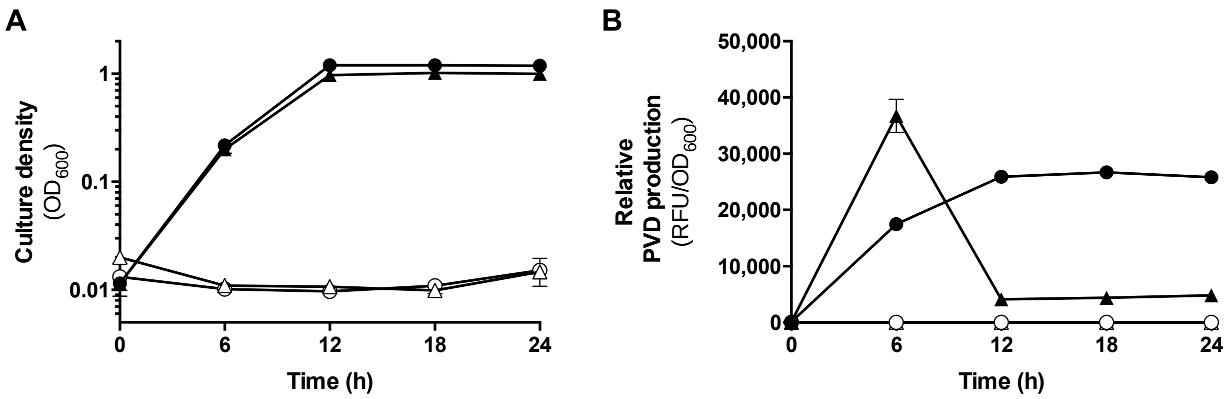

Supplementary Fig. 2. Time-courses of *P. aeruginosa* growth and PVD production. (A) Culture density, measured as optical density at 600 nm (OD_{600}). (B) Relative PVD production, expressed as relative fluorescence units (RFU) per OD_{600}. Individual cultures of the WT (filled symbols) and *pvdS* mutant (open symbols) were grown for 24 h in either C-MEM (circles) or P-MEM (triangles). Error bars show SEM and are too small to be seen in some cases, n=3.

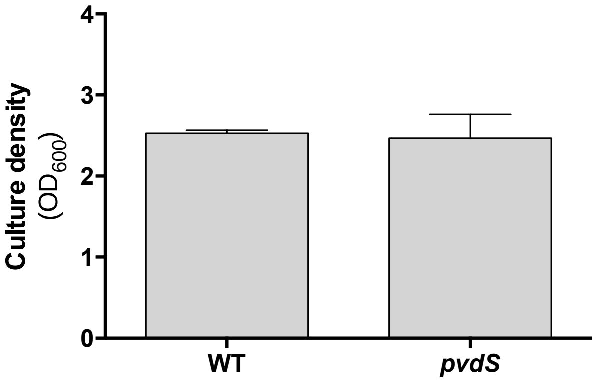

Supplementary Fig. 3. Growth yield of *P. aeruginosa* in Fe-replete batch culture. Individual cultures of the WT and *pvdS* mutant were grown for 24 h in MEM containing 20 mM C (glucose), 10 mM P, and 50 µM Fe. Culture density was measured as optical density at 600 nm (OD_{600}). Error bars show SEM, n=3. Means are not significantly different as determined by two sample t-test ($p > 0.05$).

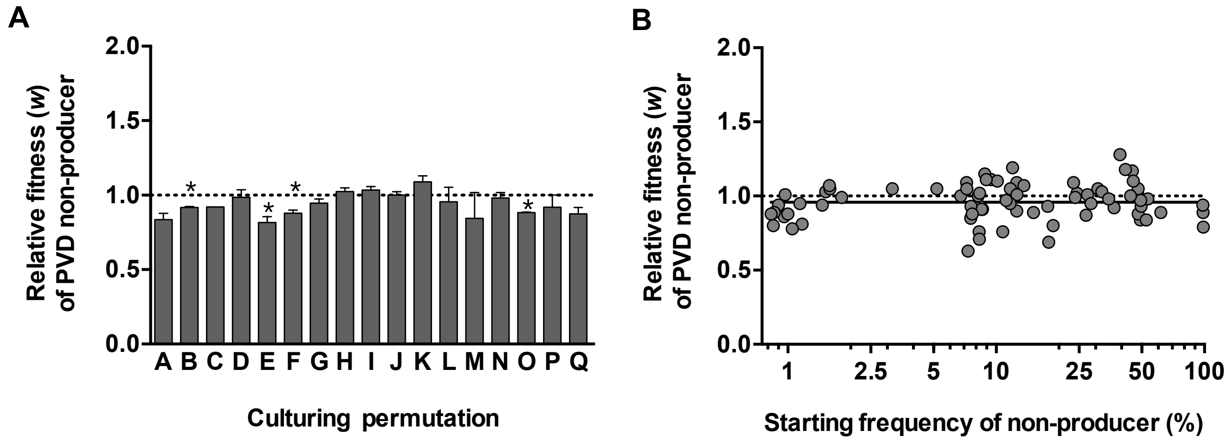

Supplementary Fig. 4. Relative fitness of PVD non-producers in a variety of batch culture permutations. (A) Relative fitness values grouped by the 17 different co-culturing permutations (see Supplementary Table 2). * indicates significant difference from 1, as determined by one sample t-test ($p < 0.05$). In no cases was fitness greater than 1. Error bars indicate SEM. (B) Individual relative fitness values as a function of starting frequency of non-producer. Each individual replicate from all 17 permutations was plotted and analyzed for frequency-dependent fitness patterns. Solid line represents linear regression of data with a slope that is not significantly different from 1 (one-sample t-test, $p > 0.05$), indicating no increase or decrease in fitness.

Supplementary Tables

Supplementary Table 1. PVD biosynthesis reactions added to the *P. aeruginosa* whole-genome metabolic model

Protein name	Activity	Reaction equation*
Precursor reactions		
PvdA	L-ornithine N5-oxygenase	L-Orn + O_2 + 2NADPH + H^+ $\rightarrow$ L-OHOrn + NADP + H_2O
PvdF	Transformylase	L-OHOrn + For + ATP $\rightarrow$ L-fOHOrn + ADP + P_i
PvdH	Aminotransferase	Already in the model
Peptide synthesis		
PvdL†	NRPS‡	L-Glu + L-Tyr + L-Dab + 3 ATP $\rightarrow$ L-Glu-D-Tyr-L-Dab + 3 AMP + 3 PP_i + 3H^+
PvdI	NRPS	L-Glu-D-Tyr-L-Dab + L-Ser + L-Arg + L-Ser + L-fOHOrn + 4 ATP $\rightarrow$ L-Glu-D-Tyr-L-Dab-D-Ser-L-Arg-D-Ser-LFOHORN + 4 AMP + 4 PP_i + 4 H^+
PvdJ	NRPS	L-Glu-D-Tyr-L-Dab-D-Ser-L-Arg-D-Ser-L-fOHOrn + L-Lys + L-fOHOrn + 2 ATP $\rightarrow$ L-Glu-D-Tyr-L-Dab-D-Ser-L-Arg-D-Ser-L-fOHOrn-L-Lys-L-fOHOrn + 2 AMP + 2 PP_i + 2 H^+
PvdD	NRPS	L-Glu-D-Tyr-L-Dab-D-Ser-L-Arg-D-Ser-L-fOHOrn-L-Lys-L-fOHOrn + L-Thr + L-Thr + 2 ATP $\rightarrow$ L-Glu-D-Tyr-L-Dab-D-Ser-L-Arg-D-Ser-L-fOHOrn-L-Lys-L-fOHOrn-L-Thr-L-Thr + 2 AMP + 2 PP_i + 2 H^+
Secretion§		
PvdE/PvdRT-OpmQ	Membrane transport	PVD + 3 ATP $\rightarrow$ PVD(e) + 3 ADP + 3 P_i + 3 H^+

* Standard abbreviations for molecules and biochemical compounds are used. Orn, ornithine; fOHOrn, N_5-formyl-N_5-hydroxyornithine; Dab, 2,4-aminobutyrate; PVD(e), extracellular (secreted) PVD.

† Initial amino acid acylation via CoA-ligase is considered energetically in the PvdL reaction above. In terms of biomass, the acyl group is again removed prior to secretion and is therefore not lost from the cell.

‡ NRPS, non-ribosomal peptide synthetase

§ Periplasmic maturation is not considered here. The roles of periplasmic enzymes Pvd N, M, O, P are not clear, and the enzymes involved in chromophore formation have yet to be identified. Consequently, the energy demand, if any, is not known. Therefore, the product of the last NRPS (PvdD), is considered to be the mature, intracellular PVD. Transport of PVD across cytoplasmic membrane by PvdE, and across outer membrane by PvdRT-OpmQ are combined here, because the model does not separately consider a periplasmic compartment. The precise stoichiometry of ATP per PVD molecule is not known, but we estimate 2 ATP for PvdE and 1 ATP for PvdRT-OpmQ.

Supplementary Table 2. Characterization of PVD producer and non-producer co-culture permutations

Group	Relative fitness (*w*)*†	p-value‡	Initial % of PVD non-producer†	n§	Starting density, (CFU mL^{-1})	Growth medium plus Fe chelator¶	Temp (°C)	Shaken (+) or static (-)	Strain pair *pvd*$^{+}$	Strain pair *pvd*$^{-}$
A	0.84 ± 0.04	0.058	10.3 ± 1.5	3	3.55×10^{7}	CAA + EDDHA	30	+	PAO1	PAO1 Δ*pvdD* Δ*pchEF*
B	0.92 ± 0.01	0.0063	8.47 ± 0.06	3	1.66×10^{5}	CAA + EDDHA	30	+		
C	0.92	N/A	8.05	1	1.74×10^{4}	CAA + EDDHA	30	+		
D	0.99 ± 0.05	0.81	46.7 ± 9.0	3	1.04×10^{4}	CAA + EDDHA	30	+		
E	0.82 ± 0.04	0.041	14.1 ± 3.1	3	8.97×10^{4}	CAA + Transferrin	37	-		
F	0.88 ± 0.02	0.0077	43.5 ± 5.5	4	6.36×10^{4}	CAA + Transferrin	37	-		
G	0.95 ± 0.03	0.074	1.40 ± 0.17	13	1.08×10^{5}	CAA + EDDHA	30	+	PAO1	PAO1 Δ*pvdS*:: Gm^{R}
H	1.03 ± 0.02	0.50	5.97 ± 0.78	2	5.10×10^{4}	CAA + EDDHA	30	+		
I	1.04 ± 0.02	0.19	11.4 ± 0.8	11	1.64×10^{5}	CAA + EDDHA	30	+		
J	1.00 ± 0.02	0.95	28.1 ± 1.9	7	7.00×10^{4}	CAA + EDDHA	30	+		
K	1.09 ± 0.04	0.064	44.6 ± 2.1	8	2.24×10^{5}	CAA + EDDHA	30	+		
L	0.95 ± 0.10	0.68	10.4 ± 2.5	4	1.92×10^{5}	25 mM Glucose + EDDHA	30	+		
M	0.84 ± 0.17	0.46	9.20 ± 1.42	3	2.10×10^{5}	25 mM Succinate + EDDHA	30	+		
N	0.98 ± 0.04	0.63	8.60 ± 0.89	4	1.97×10^{5}	25 mM Glutamate + EDDHA	30	+		
O	0.88 ± 0.01	0.00080	0.900 ± 0.054	3	6.55×10^{6}	CAA + EDDHA	30	+	PAO1 Tp^{R}	PAO1 Δ*pvdS* Tet^{R}
P	0.92 ± 0.08	0.50	48.4 ± 4.2	2	5.76×10^{6}	CAA + EDDHA	30	+		
Q	0.84 ± 0.05	0.10	98.4 ± 0.2	3	4.88×10^{6}	CAA + EDDHA	30	+		

N/A, not applicable
* Relative fitness (*w*) of PVD non-producer strain
† Values are given as the average ± SEM
‡ p-value of one-sample t-test to assess whether fitness values are significantly different than 1
§ Number of biological replicates for corresponding permutation
¶ All media shared low iron salt base (see *Supplementary methods*), with specific C-source as indicated; CAA, 0.5% w/v casamino acids

Supplementary Note 1

PVD production is immune to cheater invasion in a wide variety of batch culture conditions. In the main text, we report that the *pvdS* mutant has a relative fitness equal to, but not greater than, 1 when co-cultured with the WT in C-MEM and P-MEM batch cultures (Fig. 3A). This outcome was strikingly similar to a collection of related co-culture experiments we had conducted using growth conditions modeled after previous studies. Generally, the experimental approach entails batch cultures with casamino acids (CAA) medium[1-8]. CAA are derived from hydrolyzed casein protein, providing a mixture of carbon and nitrogen in the form of amino acids and small peptides. We present our culturing data in Supplementary Fig. 4 and associated Supplementary Table 2, where the relative fitness of non-producing strains was measured in a total of 17 different permutations.

Within this data set, we manipulated variables that could plausibly impact the relative fitness of a non-producer. Some variables tested are specifically predicted to impact the stability of cooperation, such as starting population density and initial mutant frequency[7-10]. Others were manipulated to exactly reflect conditions in other studies, such as the temperature, iron chelator of choice, and whether cultures were incubated statically or with shaking[3-5]. We also carefully considered the specific genetic mutations conferring the non-producer phenotype, and explored several different defined non-producer/producer strain pairs. A few permutations also contained glucose, succinate and glutamate as alternative C sources. However, we collectively found that these variations had little influence, as the relative fitness of non-producers was always equal to or below 1 (Supplementary Fig. 4 and Supplementary Table 2).

We found this outcome to be intriguing. Although non-producers could restore their individual growth by utilizing the PVD secreted by the WT, they did not have a selective advantage. These results began to question the social cheating hypothesis as an explanation for the evolution of PVD-negative isolates observed in nature. Furthermore, our findings appear to contrast previous descriptions of social

cheating which use conditions similar or even identical to those we explored (Supplementary Fig. 4 and Supplementary Table 2)[1-8]. Although it is not entirely clear how to reconcile our results with the previous literature, we believe that two experimental design choices in particular can at least partially explain this discrepancy.

The first such design choice is the use of non-isogenic PVD-producer/non-producer strain pairs that differ by mutations other than that conferring the PVD phenotype. In fact, many studies, including some of the first and most influential reports of social cheating, used such strains[1-3, 7, 8, 11, 12]. A prominent strain pair used was *P. aeruginosa* PAO1, a PVD producer, and PAO6609, a PVD-deficient mutant derived by UV-mutagenesis[1]. PAO6609 carries at least four mutations, *met-9011, amiE200, strA,* and *pvd9*, compared to the PAO1 parent strain. These mutations, uncharacterized by sequence analysis, confer methionine auxotrophy, amidase-deficiency and streptomycin-resistance, in addition to PVD deficiency[13, 14].

We note that the use of non-isogenic or undefined strains does not by definition negate these studies. However, it complicates the process of attributing an observed fitness outcome to the PVD phenotype, as one cannot exclude the possibility that other, possibly unknown mutations contribute to the results. Fortunately, a number of studies have been published that also employ isogenic producer/non-producer pairs[2, 3, 7, 8, 15]. These studies indicate that non-isogenic PVD non-producers tend to have higher relative fitness values compared to their isogenic counterparts, which often only have a marginal fitness advantage, if any. It therefore seems that additional mutations present in non-isogenic PVD non-producers contributed to results in early studies. Whether these putative mutations are a prerequisite of the cheating phenotype, or would confer a fitness benefit independent of siderophore cooperation is unclear. Our findings presented in the main text largely suggest that the batch-culturing format is not conducive to social cheating. However, it may indeed be that these strains are capable of

cheating in batch culture conditions, but that the field has not recognized additional important underlying mutations supporting this behavior.

A second possible explanation for the discrepancy could be variation in the growth media itself. Batches of commercially available hydrolyzed caseinate can differ in their composition, and different sources of CAA have even been shown to stimulate different PVD production phenotypes[16]. It is therefore plausible that our media preparations differed from those of previous authors, which further emphasizes the value of defined growth media.

Together with the data presented in the main text, we are led to conclude that batch culture formats, including when CAA is used as a growth substrate, do not create selective pressures conducive to social cheating. We have outlined some plausible explanations that may account for the discrepancies between our findings and those of others, although the definitive cause remains unclear.

Supplementary Methods

Bacterial growth conditions for undefined batch culture experiments. For all co-culture experiments presented in Supplementary Fig. 4 and Supplementary Table 2, bacterial strains were routinely grown to stationary phase in LB, washed in a low iron salts base and mixed to desired ratios before inoculating co-cultures. For all permutations, we used 0.118 % (w/v) $K_2HPO_4 \cdot 3H_2O$, 0.025% (w/v) $MgSO_4 \cdot 7H_2O$ as a low Fe salt base, following previous precedent[1-6, 8]. As pH can impact PVD properties and Fe solubility, we also buffered all media to a pH of 7 with 25 mM MOPS[17-19]. Where indicated, media were supplemented with 0.5% (w/v) CAA, which provides enough C and N to achieve final densities of ~ 1.25×10^9 CFU/ml. In conditions L, M, and N, glucose, succinate or glutamate was added instead of CAA, to a final concentration of 25 mM. Because N is not available in glucose, and to a lesser degree in succinate and glutamate (compared to CAA), 25 mM NH_4Cl was added to these conditions. In all conditions, 1 µM $FeCl_3 \cdot 6H_2O$ was added. The Fe chelator EDDHA (Complete Green Company, El Segundo, CA) was added to a

final concentration of 0.5 mg/ml. In some cases, 100 µg/ml apo-transferrin (Sigma) replaced EDDHA as the Fe chelator as indicated.

Bacterial strains and method of differentiation used in undefined batch culture experiments. All strains described in Supplementary Table 2 are derived from the PAO1 wild-type (ATCC 15692). PAO1 Δ*pvdD* Δ*pchEF* and PAO1 Δ*pvdS*::Gm^R were generously provided by Dr. Michael Vasil[20, 21]. PAO1 Δ*pvdS* was constructed by splicing-by-overlap-extension PCR as described in the main text. Trimethoprim and tetracycline resistance markers were chromosomally integrated to create PAO1 Tp^R and PAO1 Δ*pvdS* Tet^R, respectively, using the miniTn7 system[22]. PVD producers and non-producers were differentiated using a selection scheme compatible with their respective genotype. Strains lacking antibiotic markers were differentiated by phenotypic screening on Kings B medium based on the characteristic green colony morphology of PVD producers[23]. Trimethoprim, gentamicin or tetracycline was used at 100 µg/ml to select for PAO1 Tp^R, PAO1 Δ*pvdS*::Gm^R or PAO1 Δ*pvdS*::Tet^R, respectively. Relative fitness (*w*) was subsequently calculated as the ratio of average growth rates (μ_{pvdS}/μ_{WT}), as described[24].

Supplementary References

1. Griffin, A.S., West, S.A. & Buckling, A. Cooperation and competition in pathogenic bacteria. *Nature* **430**, 1024-1027 (2004).
2. Jiricny, N. *et al.* Fitness correlates with the extent of cheating in a bacterium. *J Evol Biol* **23**, 738-747 (2010).
3. Kummerli, R. & Brown, S.P. Molecular and regulatory properties of a public good shape the evolution of cooperation. *Proc Natl Acad Sci USA* **107**, 18921-18926 (2010).
4. Kummerli, R., Gardner, A., West, S.A. & Griffin, A.S. Limited dispersal, budding dispersal, and cooperation: an experimental study. *Evolution* **63**, 939-949 (2009).
5. Kummerli, R., Griffin, A.S., West, S.A., Buckling, A. & Harrison, F. Viscous medium promotes cooperation in the pathogenic bacterium *Pseudomonas aeruginosa*. *Proc Biol Sci* **276**, 3531-3538 (2009).
6. Kummerli, R., Jiricny, N., Clarke, L.S., West, S.A. & Griffin, A.S. Phenotypic plasticity of a cooperative behaviour in bacteria. *J Evol Biol* **22**, 589-598 (2009).
7. Ross-Gillespie, A., Gardner, A., Buckling, A., West, S.A. & Griffin, A.S. Density dependence and cooperation: Theory and a test with bacteria. *Evolution* **63**, 2315-2325 (2009).

8. Ross-Gillespie, A., Gardner, A., West, S.A. & Griffin, A.S. Frequency dependence and cooperation: Theory and a test with bacteria. *Am Nat* **170**, 331-342 (2007).
9. Darch, S.E., West, S.A., Winzer, K. & Diggle, S.P. Density-dependent fitness benefits in quorum-sensing bacterial populations. *Proc Natl Acad Sci USA* **109**, 8259-8263 (2012).
10. West, S.A., Griffin, A.S., Gardner, A. & Diggle, S.P. Social evolution theory for microorganisms. *Nat Rev Microbiol* **4**, 597-607 (2006).
11. Brockhurst, M.A., Buckling, A., Racey, D. & Gardner, A. Resource supply and the evolution of public-goods cooperation in bacteria. *BMC Biol* **6** (2008).
12. Kummerli, R., van den Berg, P., Griffin, A.S., West, S.A. & Gardner, A. Repression of competition favours cooperation: experimental evidence from bacteria. *J Evol Biol* **23**, 699-706 (2010).
13. Meyer, J.M., Neely, A., Stintzi, A., Georges, C. & Holder, I.A. Pyoverdin is essential for virulence of *Pseudomonas aeruginosa*. *Infect Immun* **64**, 518-523 (1996).
14. Rella, M., Mercenier, A. & Haas, D. Transposon insertion mutagenesis of *Pseudomonas aeruginosa* with a Tn5 derivative - Application to physical mapping of the Arc gene-cluster. *Gene* **33**, 293-303 (1985).
15. Harrison, F. Dynamic social behaviour in a bacterium: *Pseudomonas aeruginosa* partially compensates for siderophore loss to cheats. *J Evol Biol* **26**, 1370-1378 (2013).
16. Kummerli, R. & Ross-Gillespie, A. Explaining the sociobiology of pyoverdin producing *Pseudomonas*: A comment on Zhang and Rainey (2013). *Evolution* **68**, 3337-3343 (2014).
17. Hider, R.C. & Kong, X.L. Chemistry and biology of siderophores. *Nat Prod Rep* **27**, 637-657 (2010).
18. Lujan, A.M., Gomez, P. & Buckling, A. Siderophore cooperation of the bacterium *Pseudomonas fluorescens* in soil. *Biol Lett* **11**, 20140934 (2015).
19. Sandoz, K.M., Mitzimberg, S.M. & Schuster, M. Social cheating in *Pseudomonas aeruginosa* quorum sensing. *Proc Natl Acad Sci USA* **104**, 15876-15881 (2007).
20. Ghysels, B. *et al.* FpvB, an alternative type I ferripyoverdine receptor of *Pseudomonas aeruginosa*. *Microbiol* **150**, 1671-1680 (2004).
21. Ochsner, U.A., Johnson, Z., Lamont, I.L., Cunliffe, H.E. & Vasil, M.L. Exotoxin A production in *Pseudomonas aeruginosa* requires the iron-regulated *pvdS* gene encoding an alternative sigma factor. *Mol Microbiol* **21**, 1019-1028 (1996).
22. Choi, K.H. & Schweizer, H.P. Mini-Tn7 insertion in bacteria with single attTn7 sites: Example *Pseudomonas aeruginosa*. *Nat Protoc* **1**, 153-161 (2006).
23. King, E.O., Ward, M.K. & Raney, D.E. Two simple media for the demonstration of pyocyanin and fluorescin. *J Lab Clin Med* **44**, 301-307 (1954).
24. Lenski, R.E. Quantifying fitness and gene stability in microorganisms. *Biotechnology* **15**, 173-192 (1991).
